# Alveolar hemorrhage following thrombolysis in STEMI: Two rare case reports and review of the literature

**DOI:** 10.21542/gcsp.2025.16

**Published:** 2025-02-28

**Authors:** Ibrahim Oumarou Hamissou

**Affiliations:** Department of Cardiology, University Mohammed-V of Rabat, Morocco

## Abstract

This retrospective study, conducted in the Cardiology-B department of Ibn Sina Hospital, Rabat, analyzed 44 patients and describes two rare cases of alveolar hemorrhage occurring after thrombolysis in patients diagnosed with ST-segment elevation myocardial infarction (STEMI).

While contributing to the development of a larger registry for this rare condition, these two unique cases from our institution are noteworthy in their own right. We also surveyed the current literature to identify risk factors for alveolar hemorrhage following thrombolysis in STEMI patients to understand the underlying pathophysiological mechanisms and propose an optimal management strategy.

## Introduction

Acute coronary syndrome with ST-segment elevation myocardial infarction (STEMI) is a time-critical condition requiring immediate reperfusion. Despite significant improvements in interventional cardiology and percutaneous coronary intervention(PCI) techniques, intravenous thrombolysis remains an effective treatment option in the absence of contraindications^1,2^. However, this therapeutic strategy has potentially severe consequences, including pulmonary hemorrhage, a rare but life-threatening complication^3,4^. Due to its rarity, there is little literature on this topic, with only 23 cases reported to date in the English medical literature^5– 23^. The pathophysiology remains poorly understood, and diagnosis in clinical practice relies on a combination of clinical, radiological, and biological findings^19,24– 26^. The development of pulmonary hemorrhage following thrombolysis for STEMI remains a therapeutic challenge.

**Figure 1.**
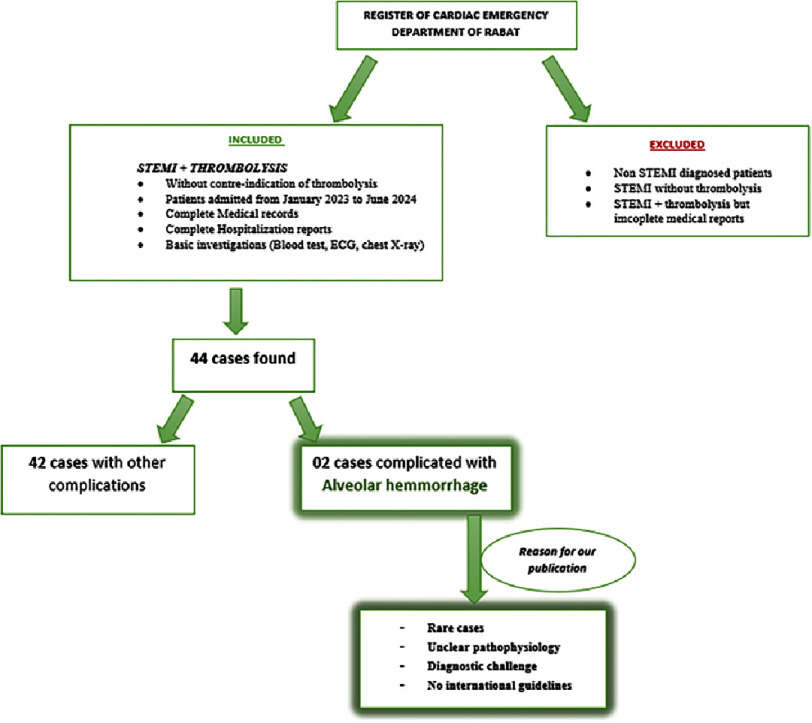
Inclusion criteria for current study.

Our methodology involved patients admitted to the emergency department for STEMI and the registry of the interventional cardiology department of Rabat. 44 patients were included, diagnosed with STEMI who underwent timely thrombolysis (in the absence of contraindications) between January 2023 and June 2024. Only cases with medical records, hospitalization reports, and at least basic investigations (blood tests, ECG, and chest X-ray) were included. Patients with a diagnosis of STEMI who did not receive thrombolysis were excluded [see Figure 1]. The data collected included patients demographic features (age and sex), their medical history, cardiovascular risk factors, the management (STEMI diagnosis, thrombolysis, coronary angiography), the occurrence of complications such as pulmonary hemorrhage (diagnosis and management), as well as prognostic factors and patient outcomes.

## CASE REPORT 1

### Medical history

The first case involved a 71-year-old man with a recent diagnosis of type-2 diabetes. He was mildly overweight (BMI=26), with a sedentary lifestyle, and a smoker. However, he had no history of hypertension, dyslipidemia, or any family history of coronary artery disease.

### History of presentation

On March 11, 2023, the day before his admission, the patient presented with retrosternal and constrictive chest pain on exertion, radiating to the scapula that spontaneously resolved within a few minutes. The following day, the patient experienced a recurrence of sudden new onset, chest pain at rest, associated with nausea, vomiting, and sweating which motivated him to report to the hospital.

**Figure 2.**
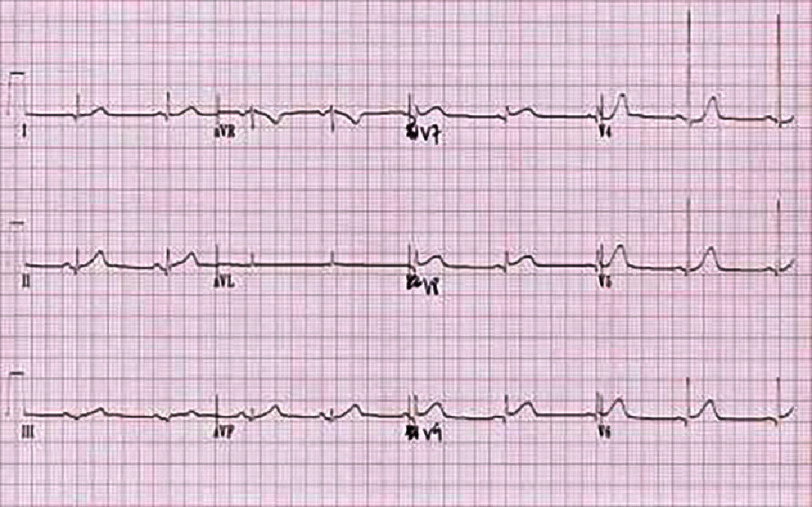
ECG of the basal leads showing a ST segment elevation more evident in the basal territory (V7, V8 and V9) in a patient in sinus rhythm.

**Figure 3.**
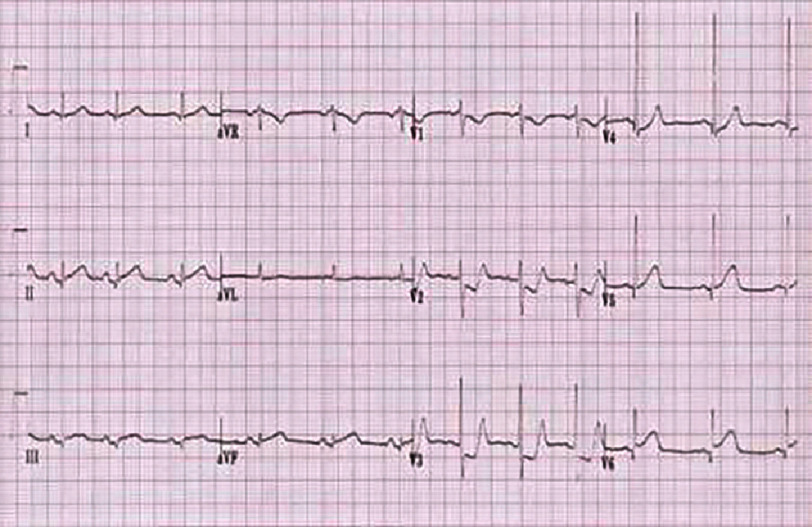
ECG of the frontal leads showing mirror images in the antero-septo-apical region in a patient in sinus rhythm.

### Investigations

The first medical contact at the emergency department was 4 h after the onset of the chest pain. An electrocardiogram (ECG) was performed within 10 min and revealed persistent ST-segment elevation (>1 mm) in the inferior, lateral, and basal leads [Figure 2], with mirror image changes in the anterior, septal, and apical leads [Figure 3], suggesting an inferio-lateral, and basal STEMI.

The patient was hemodynamically stable with a blood pressure of 140/75 mmHg in both arms, a heart rate of 77 beats per minute, he was not in respiratory distress with an oxygen saturation of 98% on room air, and had a respiratory rate of 18 cpm. Cardiac auscultation revealed normal heart sounds without any pathologic murmurs or pericardial rub. There were no signs of heart failure, and peripheral pulses were well palpable.

### Management

The patient received a loading dose of antiplatelet agents (300 mg of aspirin and 300 mg of clopidogrel) and anticoagulation with enoxaparin (0.8 cc). The decision to administer tenecteplase (Metalyse: 40 mg IV) for thrombolysis was made concurrently, 5 h from the onset of the chest pain. Monitoring showed criteria for successful thrombolysis (exacerbation followed by decreasing of pain, 50% of reduction in ST-segment elevation) but also revealed a paroxysmic complete heart block at the rate of 60 bpm, which resolved by the 90-minute mark. Minor hemoptysis was also noted during this time. The patient was subsequently transferred to the cardiology department the same day.

**Figure 4.**
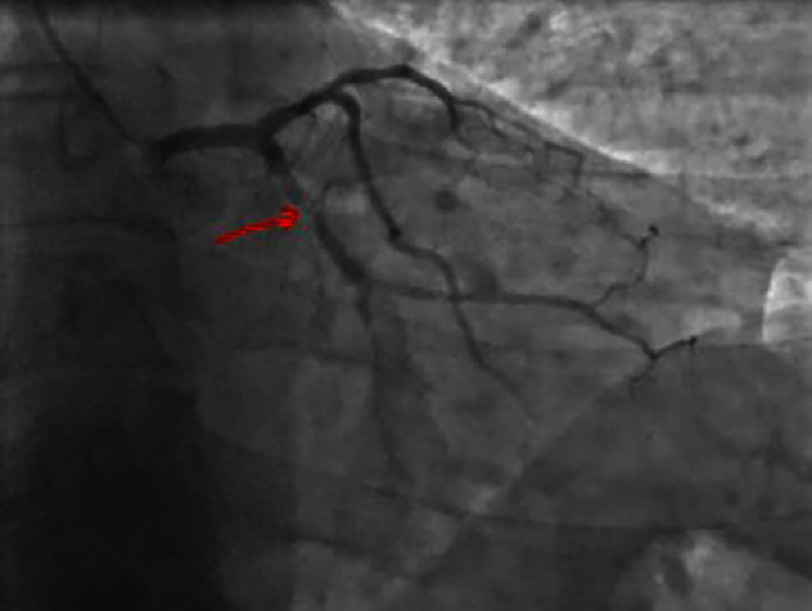
Right anterior oblique caudal view showing a significant lesion of the proximal-middle CX.

**Figure 5.**
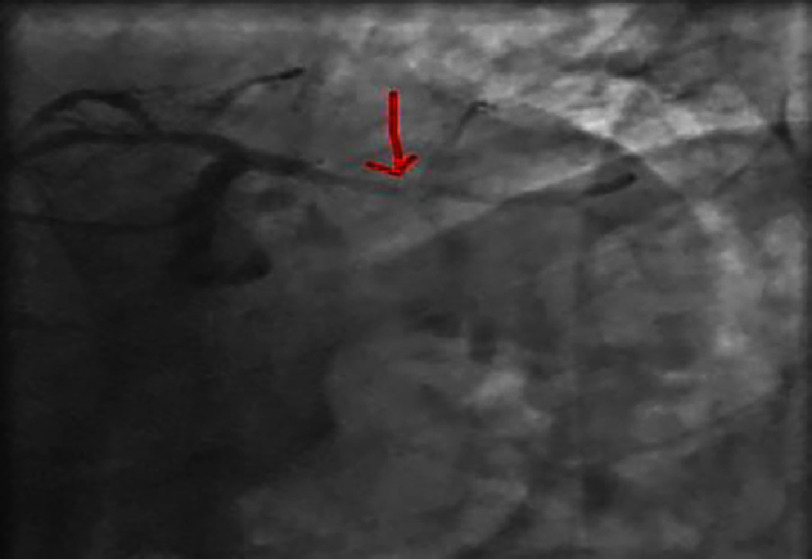
Significant lesion of the proximal-middle circumflex artery on a spider view.

**Figure 6.**
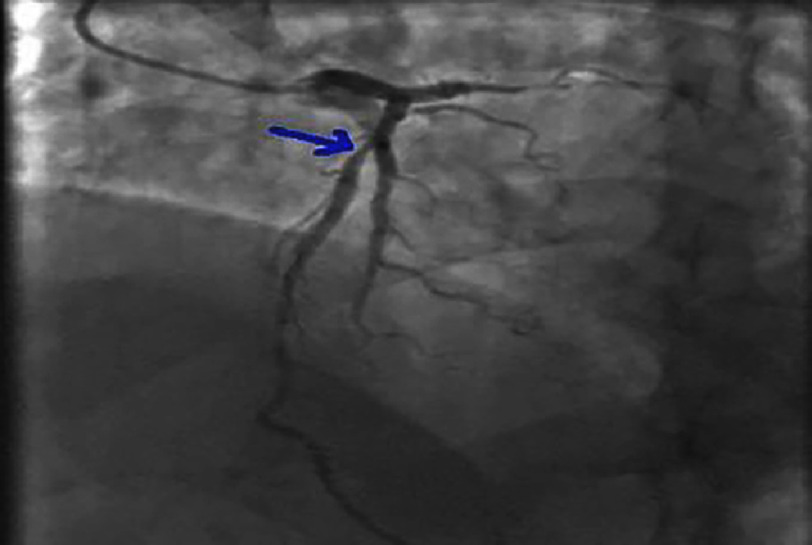
Non-significant lesion of the left anterior descending artery (LAD) distal to the second diagonal branch.

**Figure 7.**
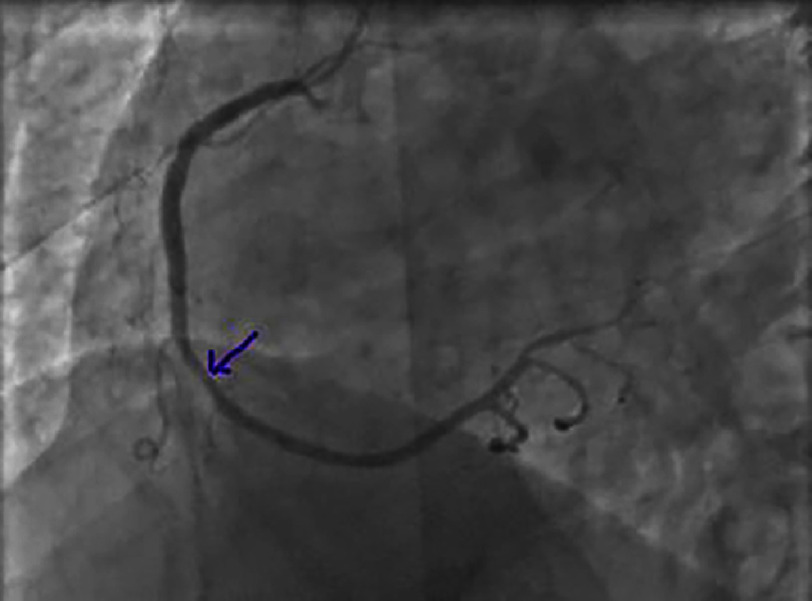
Non-significant lesion of the distal right coronary artery.

Coronary angiography confirmed a significant lesion in the proximal-mid segment of the left circumflex artery (LCX) with a TIMI flow of 3 [Figures 4 and 5]. The left anterior descending artery (LAD) and right coronary artery (RCA) were without significant lesions [Figures 6 and 7].

**Figure 8.**
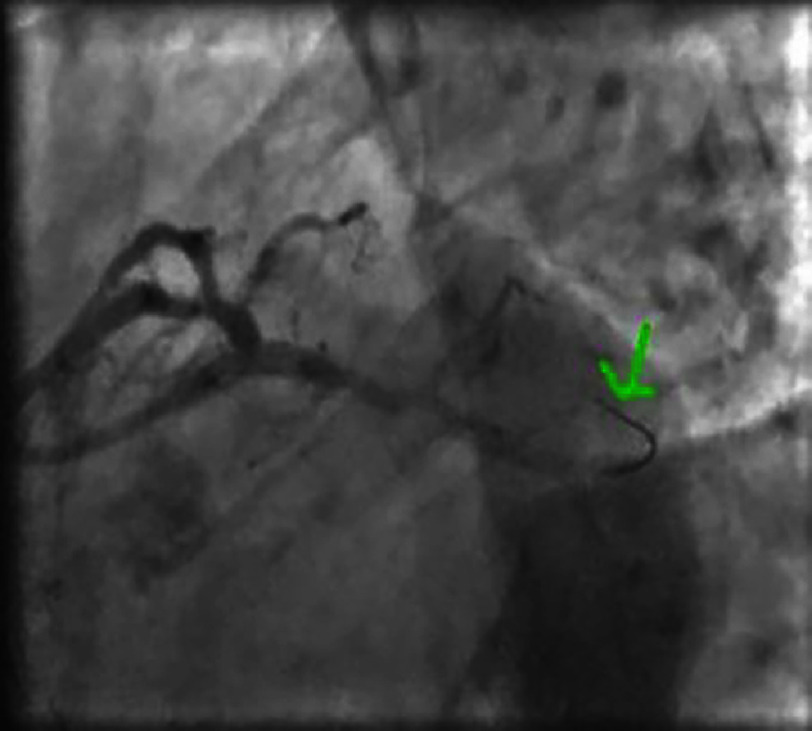
Advancement and placement of the guide wire into the distal portion of the circumflex coronary artery.

**Figure 9.**
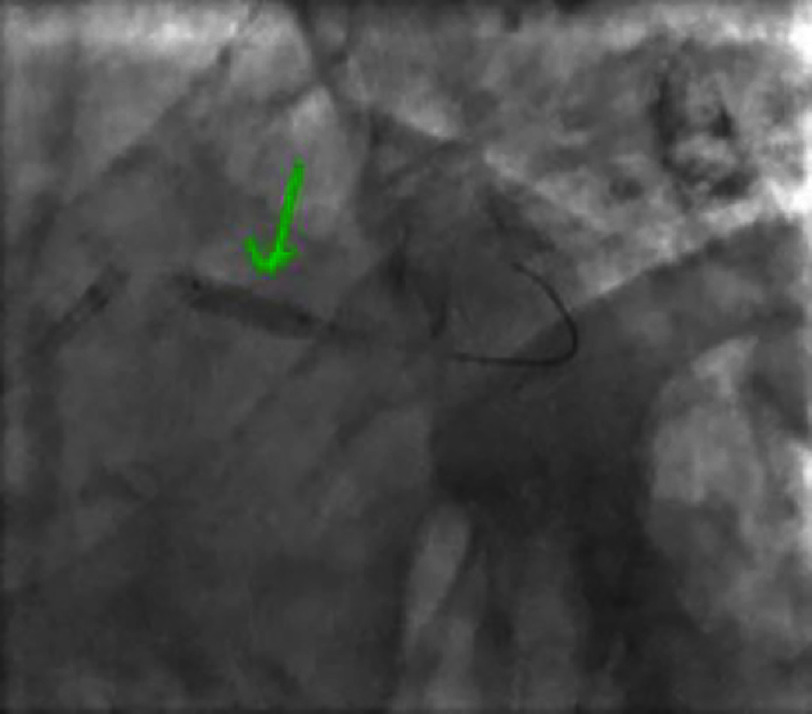
Balloon inflation at the lesion site.

**Figure 10.**
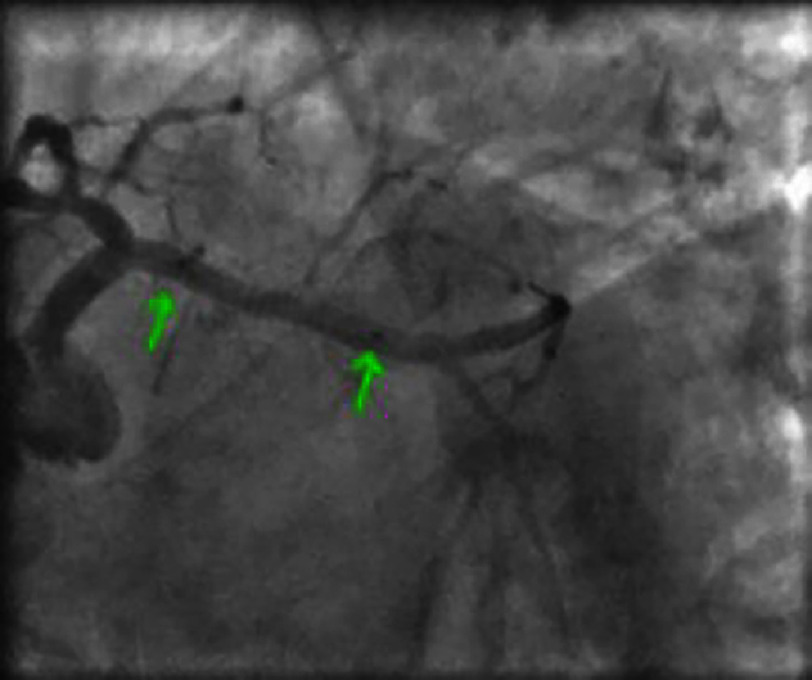
Deployment of the stent in the circumflex artery.

**Figure 11.**
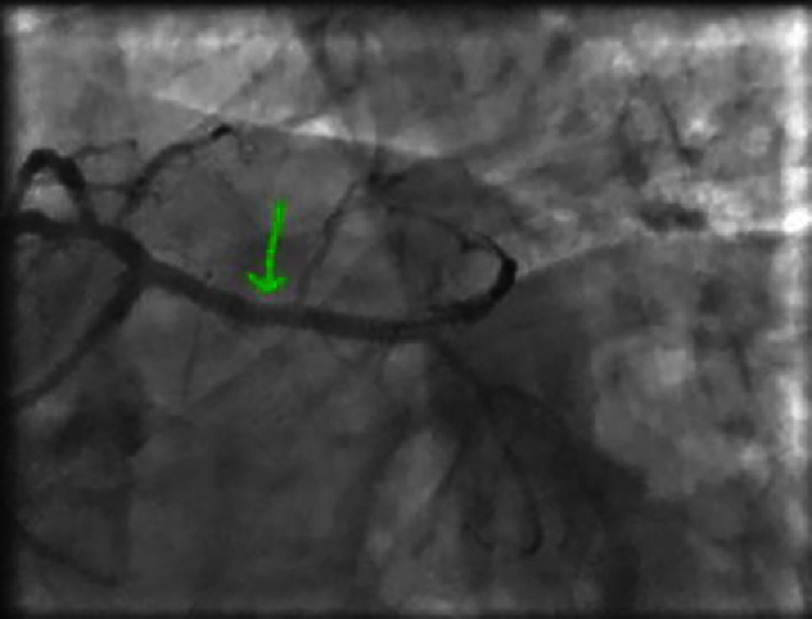
Optimal deployment of the stent in the circumflex artery with a good angiographic result (Spider view).

**Figure 12.**
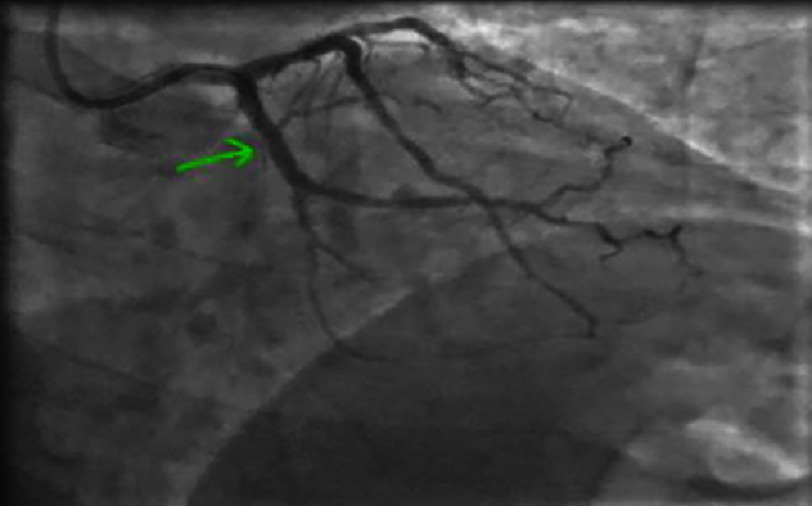
Optimal deployment of the stent in the circumflex artery with a good angiographic result (Right anterior oblique caudal view).

The procedure concluded with angioplasty of the LCX (culprit artery) and deployment of a stent [Figures 8, 9, 10, 11 and 12]. Following the procedure, the patient developed sustained ventricular tachycardia (VT) that was cardioverted to sinus rhythm and amiodarone was initiated as a continuous infusion for 24 h.

### Evolution

On the same day of admission, laboratory tests revealed an elevated troponin level of 915.78 ng/L (23 times the upper limit of normal), slightly elevated AST levels at 49 U/L (normal ALT), hyperglycemia at 3.25 g/L (HbA1c = 7.5%), and an initially normal hemoglobin level of 16.2 g/dL with normal white blood cell and platelet counts. Renal function was normal, and C-reactive protein was negative at 2.3 mg/L. Lipid profile was normal with LDL at 0.99 g/L. Coagulation studies were unremarkable (PT = 74% and aPTT = 1.03). Transthoracic echocardiography (TTE) showed a non-dilated, non-hypertrophied left ventricle with akinesia of the basal and mid segments of the inferior and inferolateral walls. Left ventricular ejection fraction (LVEF) was 50% (SBP), with elevated left ventricular filling pressures. There was no thrombus or ventricular septal defect. The right ventricle was not dilated and had good systolic function. There was no evidence of pulmonary hypertension. The pericardium was dry. The inferior vena cava was not dilated. No valvular disease was identified.

**Figure 13.**
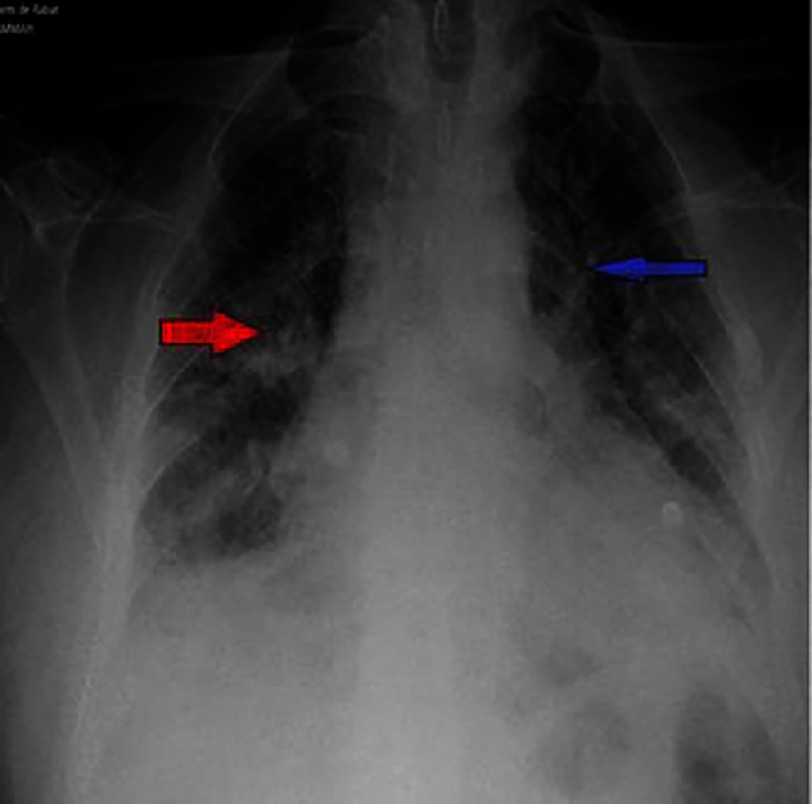
Supine posteroanterior chest X-ray showing bilateral, rounded opacities with blurred margins, predominantly in the lower two-thirds of both lung fields (red arrow), associated with linear opacities (blue arrow), suggestive of an alveolar-interstitial syndrome.

### Complications

On the second day of hospitalization, the patient developed acute dyspnea with hypoxemia (SpO2 = 87% on room air) and increased hemoptysis. Physical examination revealed bilateral crackles in the lower two-thirds of the lungs, and a chest X-ray showed an alveolar-interstitial syndrome [Figure 13].

**Figure 14.**
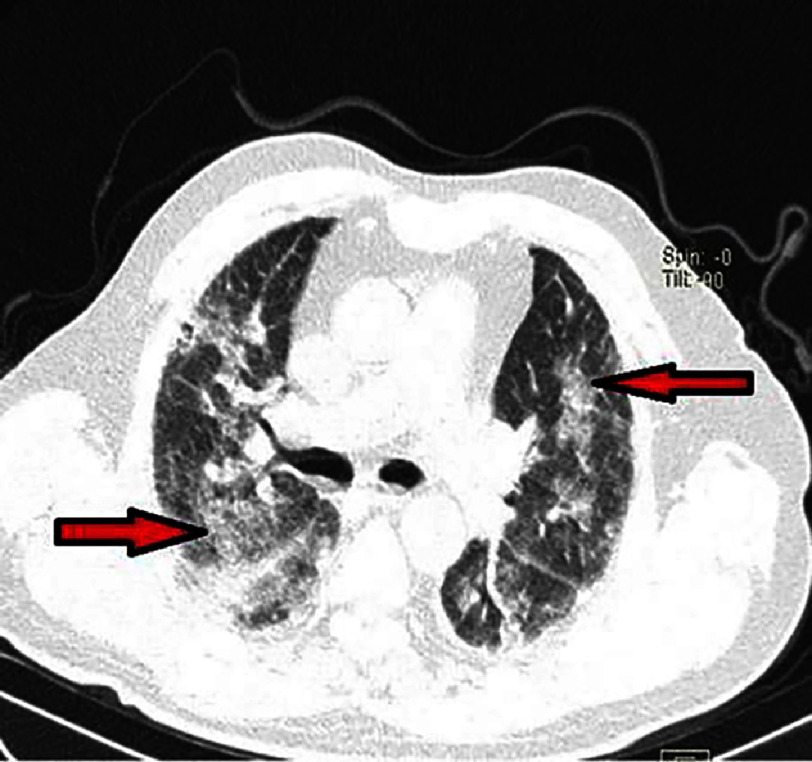
Chest CT scan showing diffuse bilateral ground-glass opacities on a lung parenchyma window (Patient #1).

**Figure 15.**
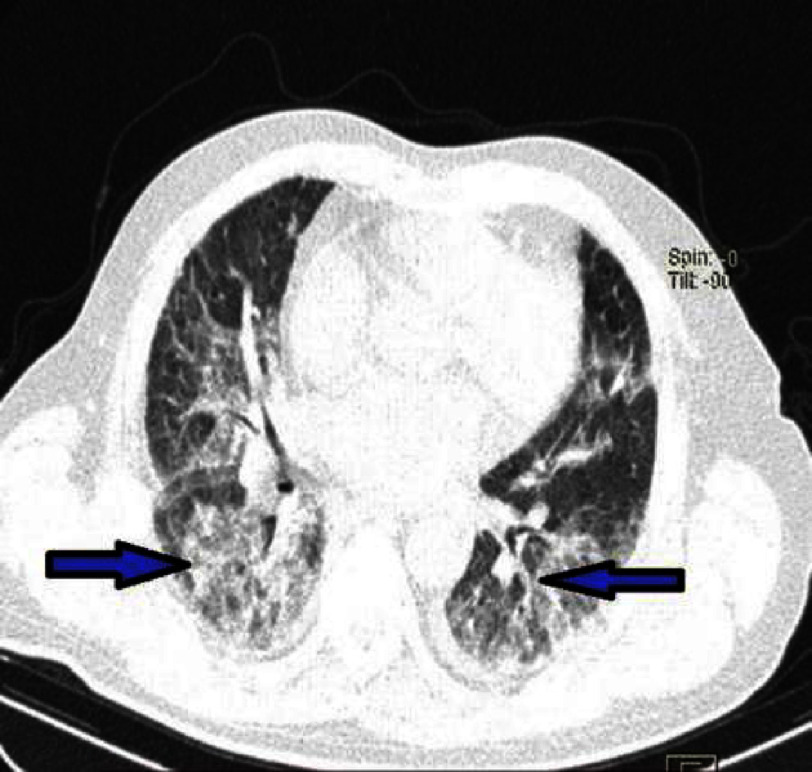
Chest CT scan showing predominantly centrilobular opacities at the bases on a lung parenchyma view (Patient #1).

**Figure 16.**
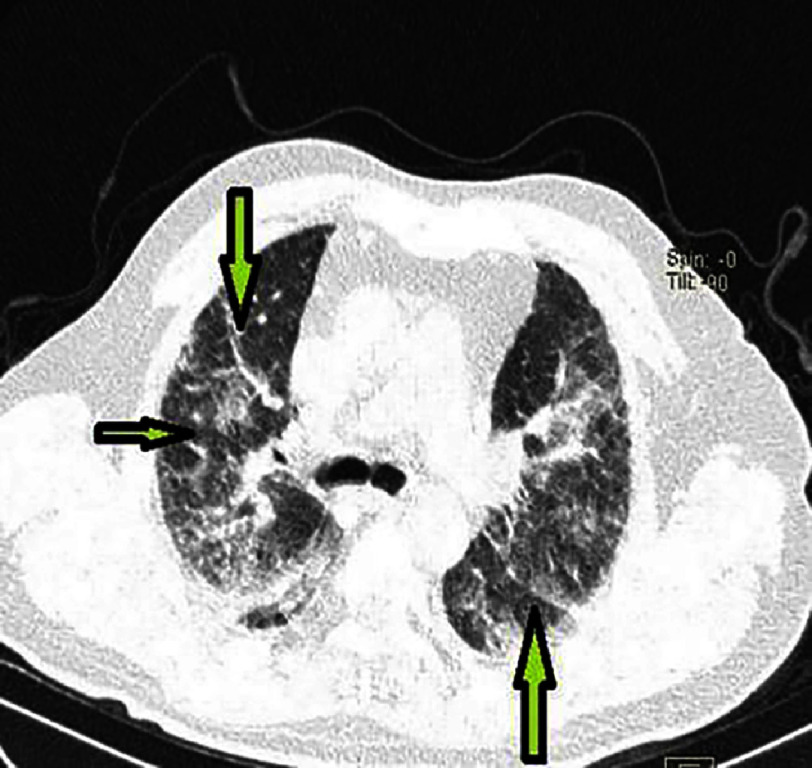
Chest CT demonstrating multiple bilateral ground-glass opacities and mild septal thickening on lung parenchyma view in patient #1.

Follow-up transthoracic echocardiography showed elevated left ventricular filling pressures but no additional kinetic abnormalities.The pericardium was dry, and there were no mechanical complications. However, a decrease in hemoglobin to 13 g/L was noted on a follow-up complete blood count. Given the increased hemoptysis, dyspnea without obvious signs of heart failure, alveolar-interstitial infiltrates on chest X-ray, and especially the drop in hemoglobin, a diagnosis of alveolar hemorrhage was suggested. A chest CT scan was highly suggestive, showing diffuse bilateral ground-glass opacities, predominantly in a centrolobular distribution with hazy borders, and associated with septal thickening [Figures 14, 15 and 16].

Consequently, after consultation with the intensivists, management consisted of stopping anticoagulation, continuing dual antiplatelet therapy, and maintaining the rest of the treatment for ischemic cardiomyopathy. Oxygen therapy was continued, and hemostatic therapy with Exacyl^®^ was initiated.

**Figure 17.**
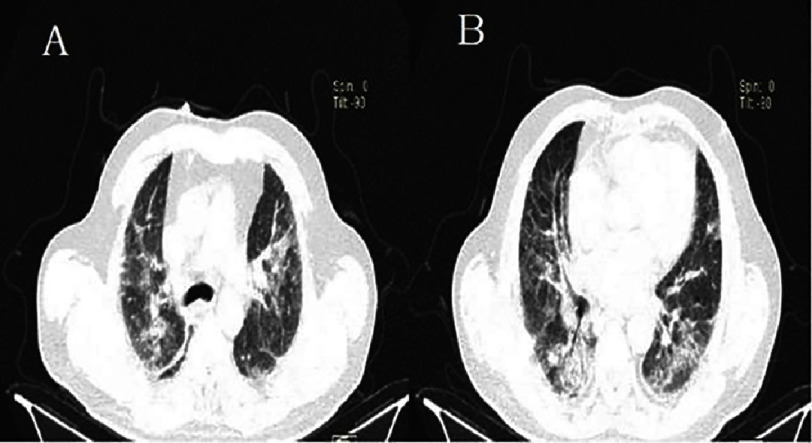
Follow-up chest CT scan on lung parenchyma window showing stable pulmonary lesions (Patient #1).

The patient’s condition progressively deteriorated, although a follow-up chest CT scan showed relatively stable pulmonary lesions (Figure 17: A and B).

**Figure 18.**
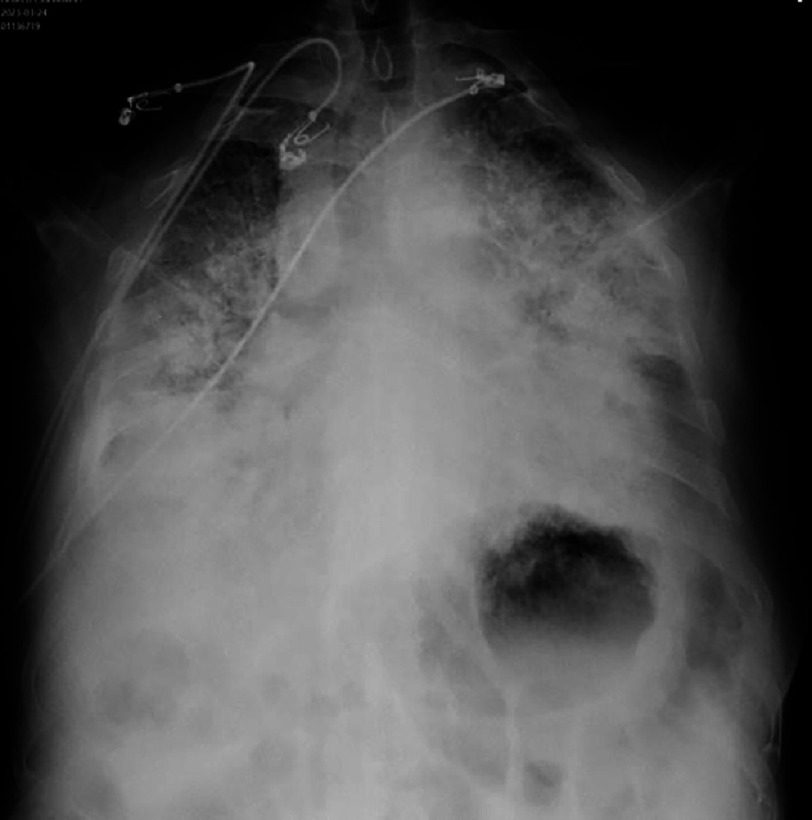
Follow-up chest X-ray, posteroanterior view, showing almost a white lung (Patient #1).

On the fifth day, the patient’s respiratory condition worsened with hypoxemia at 70% on room air, associated with purulent sputum and an increase in inflammatory markers. Chest X-ray showed almost complete whiteout of the lungs (Figure 18).

This necessitated transfer to the intensive care unit, and dual antibiotic therapy with amoxicillin and ciprofloxacin was initiated. Despite continued treatment, the patient rapidly developed severe respiratory distress followed by cardiac arrest, with a non-successful cardiopulmonary resuscitation.

## CASE REPORT 2

### Medical history

The second case involved a 65-year-old male patient who was an active smoker and had type 2 diabetes. No other medical or surgical history was found.

### History of presentation

Symptoms began on the day of admission (September 1, 2023) with a sudden onset, severe and crushing chest pain, without radiation and associated with autonomic symptoms. The patient presented to the emergency department 2 h after the onset of the pain.

### Investigation

**Figure 19.**
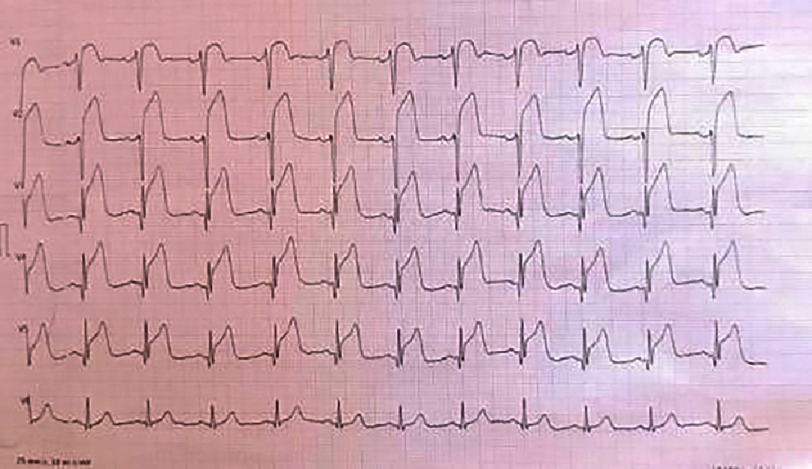
12-lead ECG showing a ST-segment elevation in the anterior leads with reciprocal changes in the inferior leads, in a patient with sinus rhythm.

**Figure 20.**
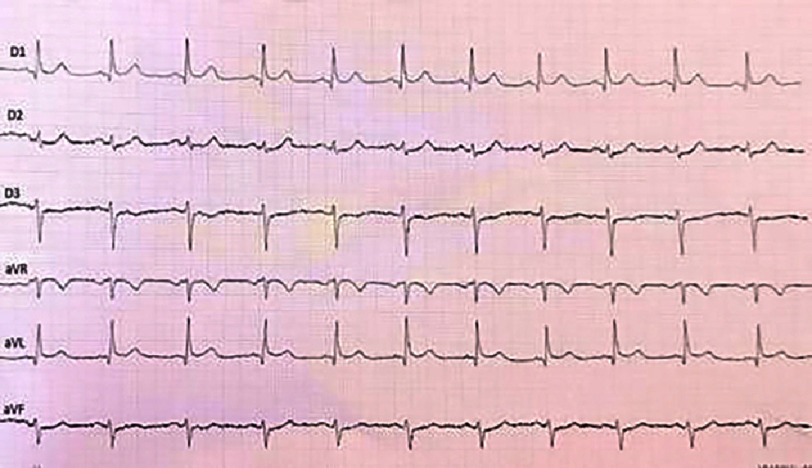
Peripheral leads (ECG) showing reciprocal changes in the inferior territory.

The initial ECG, performed 10 min after first medical contact, showed a ST-segment elevation in the anterior and high lateral leads (>two mm in V2, V3, and >one mm in other leads)], with a reciprocal change in the inferior leads, suggesting an extensive anterior STEMI [Figures 19 and 20].

### Management

The patient had initially undergone thrombolysis with tenecteplase (Metalyse/weight-based dose) which failed followed by several episodes of hemoptysis. Subsequently, he presented a cardiac arrhythmia storm with ventricular fibrillation, necessitating five direct current cardioversion (DCCV) and subsequent orotracheal intubation (after the 2nd DCCV). Approximately 30 min of cardiopulmonary resuscitation (CPR) were required to recover the patient who was in cardiogenic shock. Vasoactive drugs (norepinephrine and dobutamine) were administered, along with continuous IV lidocaine.

**Figure 21.**
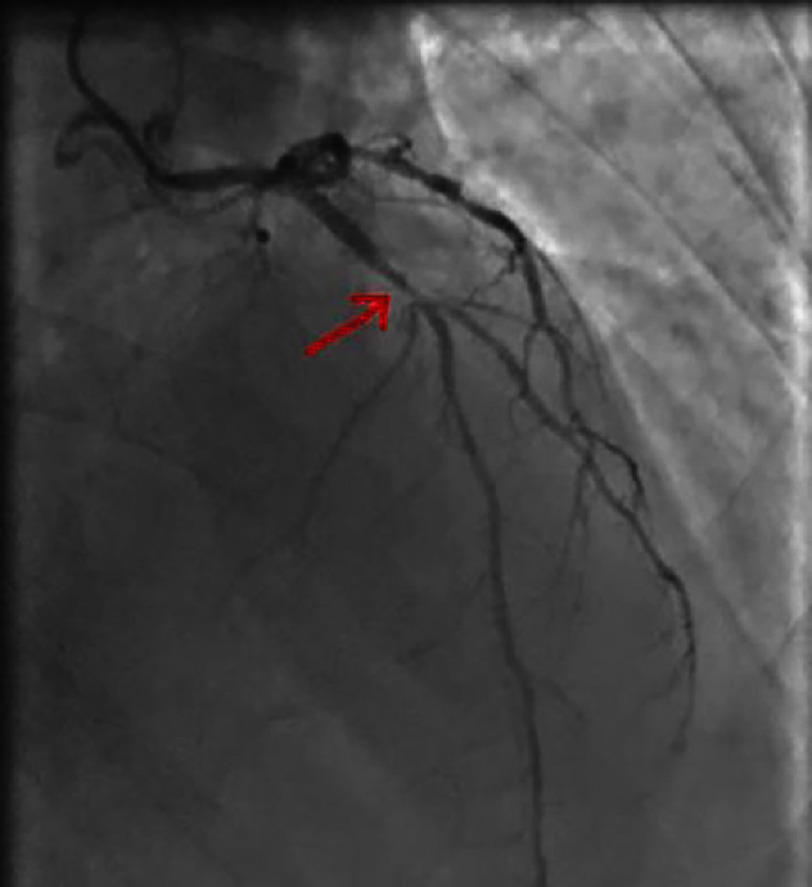
Coronary angiography showing a heterogeneous subocclusive lesion of the proximal left anterior descending artery (LAD) involving the origin of the first diagonal (Medina 1-0-1).

**Figure 22.**
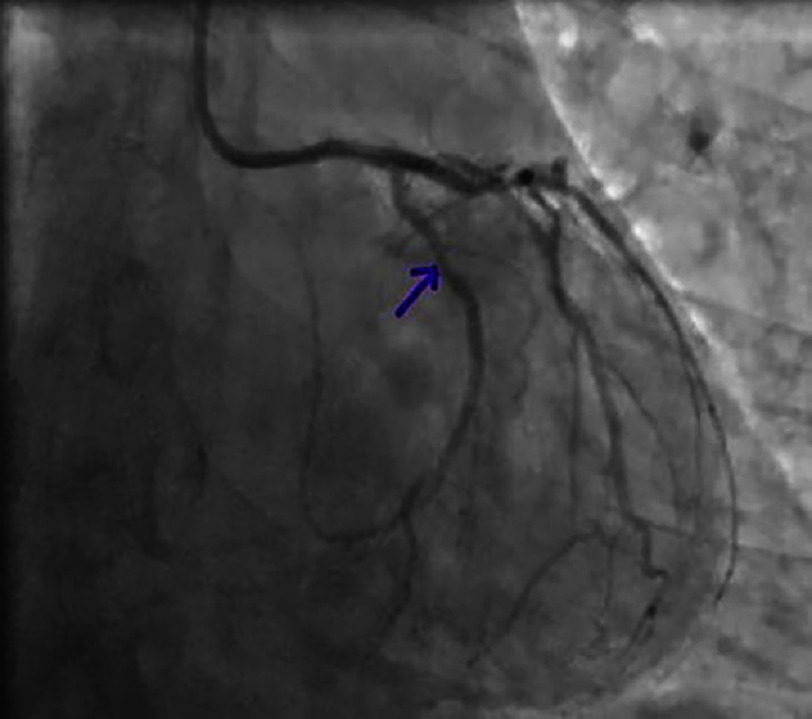
Caudal view showing a non-significant lesion of the mid circumflex artery.

**Figure 23.**
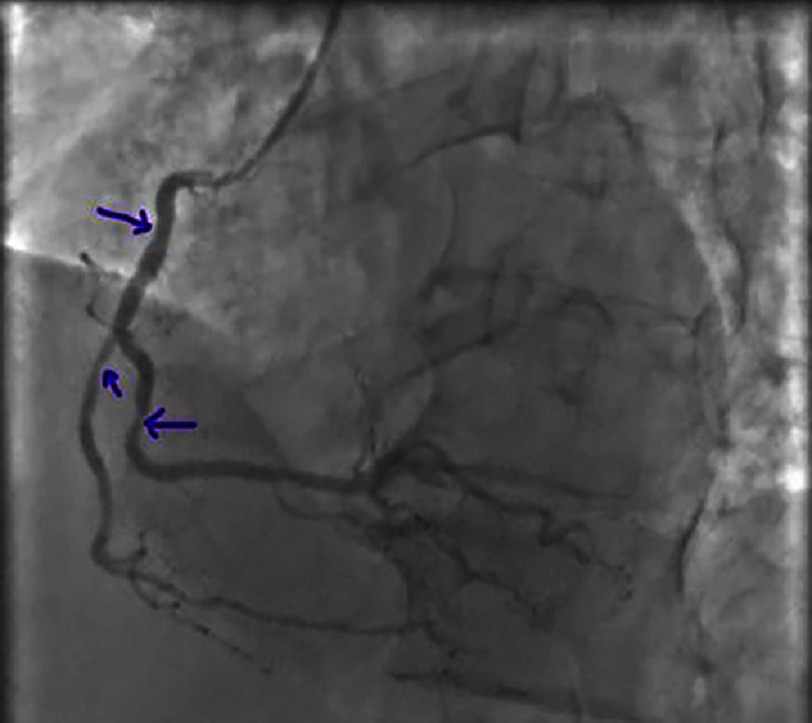
Intermediate lesions of the mid RCA), distal RCA), and marginal artery of RCA.

Subsequent coronary angiography revealed a thrombotic lesion of the proximal left anterior descending artery (LAD) involving the origin of the first diagonal (Medina 1-0-1), which was the culprit lesion for the infarction with a TIMI 3 flow [Figure 21]. The left circumflex (LCX) and right coronary artery (RCA) arteries showed non-significant lesions [Figures 22 and 23].

**Figure 24.**
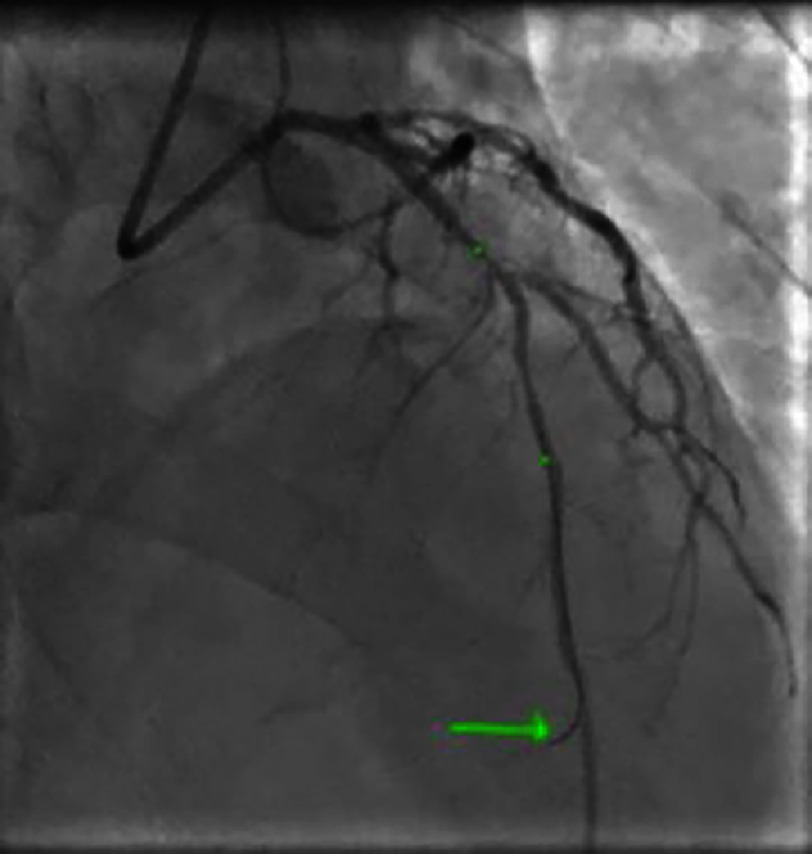
Advancement and placement of the guidewire at the distality of the left anterior descending artery.

**Figure 25.**
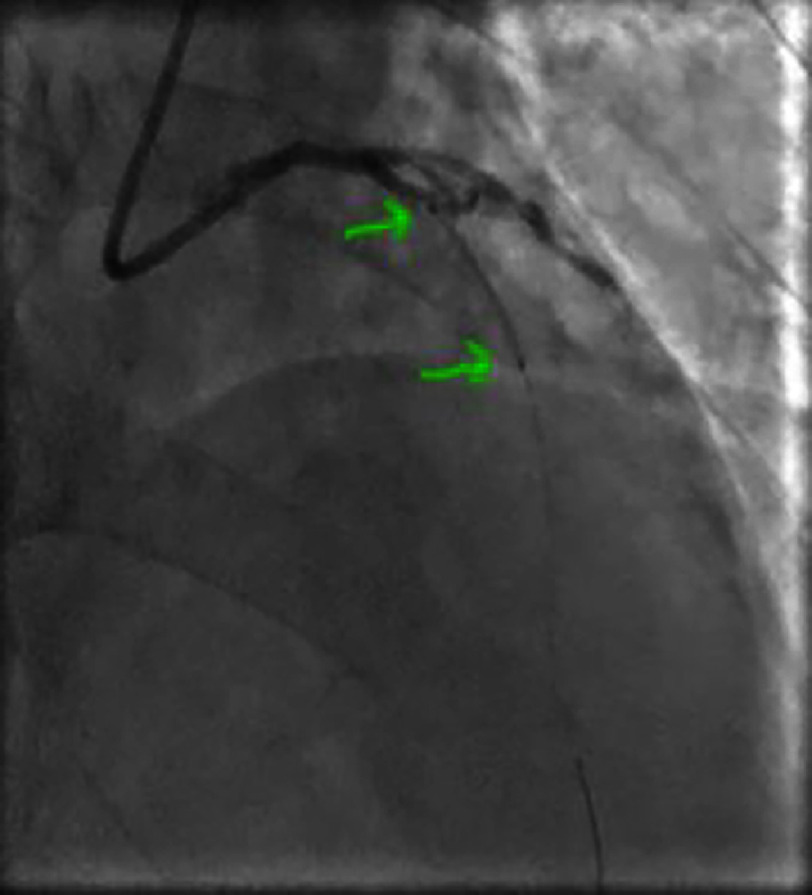
Guidewire and stent positioned at the site of the stenosis before balloon inflation.

**Figure 26.**
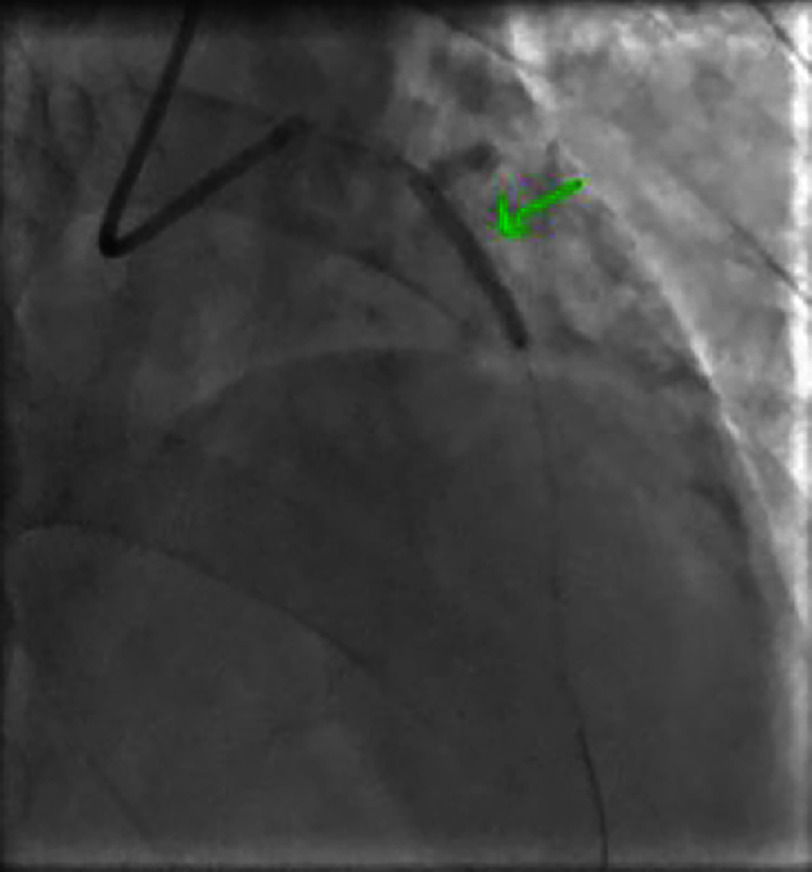
Balloon inflation and deployment of the stent at the target lesion in the LAD.

**Figure 27.**
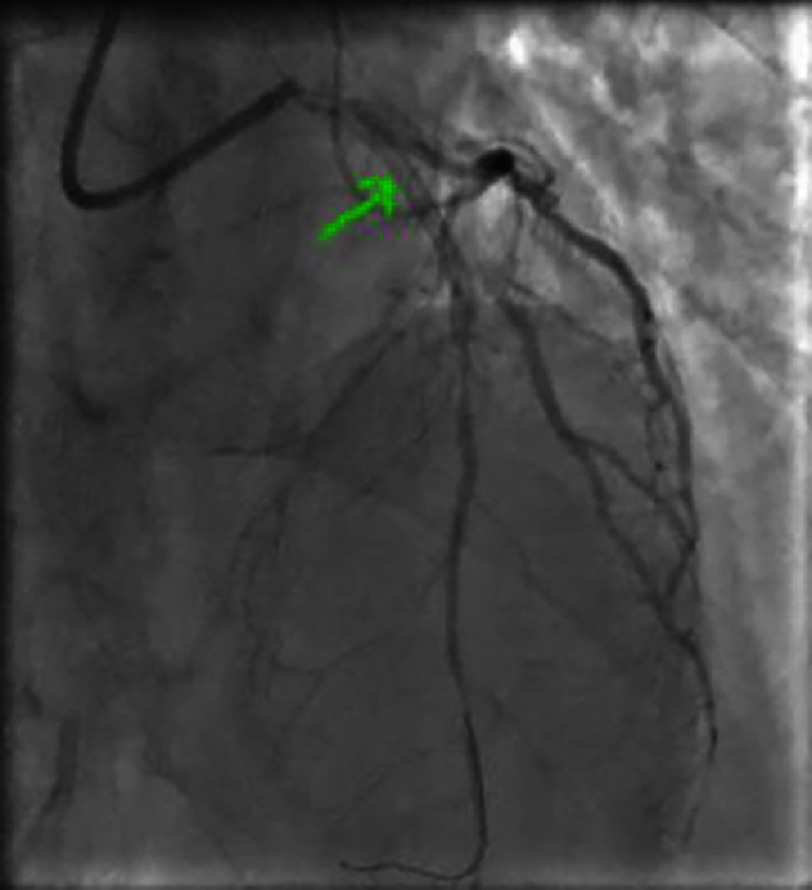
Stent deployed with the presence of a vasospasm at the stent’s distal edge and a TIMI 3 flow.

Percutaneous coronary intervention (PCI) of the LAD was performed with implantation of a stent and a good final angiographic result [Figures 24, 25, 26 and 27]. The patient was transferred to the intensive care unit on the same day.

### Evolution

**Figure 28.**
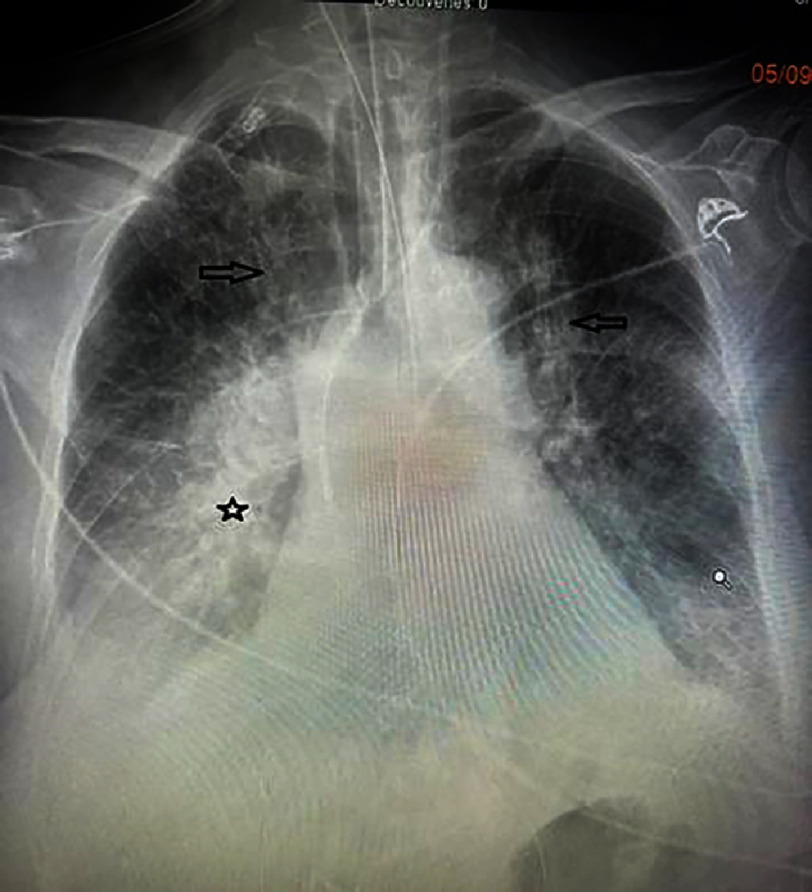
Chest X-ray (Patient #2) posteroanterior view showing an alveolo-interstitial syndrome (arrows) with a right basal focus (star).

Clinical examination on admission found an intubated and ventilated patient, sedated, with a blood pressure of 100/60 mmHg, a heart rate of 115 bpm on vasoactive drugs, and the presence of blood streaks in the oropharynx. Pulmonary auscultation revealed diffuse crackles and rhonchi without clear signs of heart failure. The ECG showed the appearance of Q waves of necrosis in the anterior leads in a patient with sinus rhythm. Transthoracic echocardiography (TTE) showed akinesis of the apex and adjacent segments of the left ventricle (LV), hypokinesia of the anterior, anteroseptal, and inferoseptal walls. The LV ejection fraction (LVEF) was 35% (SBP), right ventricular (RV) function was preserved, there were no intracavitary thrombi or mechanical complications, and the pericardium was dry. Laboratory tests revealed troponin >50,000 ng/L, an initial hemoglobin of 13.2 g/dL with elevated white blood cell count at 18,000 cells/mm3, normal platelets, an increase in creatinine to 53 mg/L, liver cytolysis (AST 1295 U/L, ALT 168 U/L), metabolic acidosis (HCO3- = 16 mEq/L), and hyperglycemia at 3.66 g/L. Coagulation studies were normal. Chest X-ray showed an alveolar-interstitial syndrome with a focus of pneumonia in the right base [Figure 28].

### Complications

On the third day, the patient’s hemodynamic and respiratory status deteriorated despite optimization of vasoactive drugs. There was significant bleeding from the oropharynx, and a major drop in hemoglobin to 10 g/dL was noted, with persistent crackles on lung auscultation despite the use of diuretics, suggesting alveolar hemorrhage as the primary diagnosis. This immediately complicated and was followed by a cardiac arrest, leading to the patient’s death.

## Literature review

### Definitions

STEMI is a time-critical emergency, most often caused by a sudden, complete, and persistent occlusion of a coronary artery, requiring rapid coronary revascularization^1^. Intravenous thrombolysis for STEMI remains an effective treatment in the absence of contraindications^2^ but has bleeding as a common side effect, including cerebral, gastrointestinal, retroperitoneal, and genitourinary bleeds^2,3,5^. Although alveolar hemorrhage is an uncommon and rare complication of intravenous thrombolysis, it is defined as bleeding occurring in the pulmonary alveolar space due to a rupture of the alveolar-capillary membrane. It is a life-threatening emergency that can lead to acute respiratory failure^3,4^.

### Epidemiology

Regarding the rarity of the case, there is no current available data in the literature concerning the incidence of alveolar hemorrhage after thrombolysis in STEMI. Only few cases have been reported in articles. Ben Mrad et al. collected just twenty cases of alveolar hemorrhage after thrombolysis in STEMI from 1990 to 2020 (excluding their two cases reported and published in 2021). We found another case report published in 2023, making a total case of 23 excluding our two cases^5– 23^.

### Physiopathology

In general, alveolar hemorrhage can be classified into three categories:

• Immune causes such as, ANCA-associated vasculitis, immune complex-mediated vasculitis with anti-basement membrane antibodies (Goodpasture’s syndrome)
• Non-immune causes such as, exposure to toxic substances (amiodarone, cannabis, silicone for cosmetic purposes), vascular trauma and
• Undetermined causes^10^.

The pathophysiology of the alveolar hemorrhage after thrombolysis in STEMI is still not well understood, however some theories have been proposed such as immune reaction due to certain molecules like streptokinase which might be responsible of capillaritis [33], Tio et al. identified some anti-steptokinase anti-bodies in patients who presented with alveolar hemorrhage after thrombolysis , which supports this theory^8^. There might exist a hypothesis based of some predisposing risk factors of alveolar hemorrhage such as anomalies of the pulmonary parenchyma, right cardiac catheterization, an arrythmia necessitating a DCCV, a presence of heart failure or a cardiopulmonary resuscitation and the use of recreative drugs such as cocaine or tobacco^22^.

### Diagnosis

The diagnosis is most commonly suspected in the presence of a triad of acute dyspnea, hemoptysis, and alveolar-interstitial infiltrates on chest X-ray associated with a drop in hemoglobin^24^. Radiographic findings are variable, initially showing diffuse alveolar opacities predominant in the perihilar regions, which may evolve into reticular opacities with either radiographic clearance after 2 weeks or persistence of sequelae interstitial opacities^25^. Chest CT most often reveals ground-glass opacities^26^. However, it is the bronchoalveolar lavage (BAL) that confirms the diagnosis by demonstrating the presence of blood or its residues^19^. The patient’s condition should be taken into account before performing such an invasive procedure.

### Management

It is also important not to confuse the alveolar hemorrhage with an acute pulmonary edema, which could delay appropriate treatment. Moreover, other etiologies should be ruled out, including ANCA-associated vasculitis, immune complex-mediated vasculitis with anti-basement membrane antibodies (Goodpasture’s syndrome), and exposure to toxic substances (amiodarone, cannabis, silicone for cosmetic purposes)^10^. This is a critical emergency, and initial management focuses on treating respiratory distress by correcting hypoxemia with oxygen therapy or positive pressure ventilation. Subsequently, blood transfusion and discontinuation of antithrombotics may be necessary, if indicated^21^.

### Evolution and prognosis

Regarding the prognosis of this complication, it depends mainly on the severity of the infarction and the depth of the hemorrhage.

## Discussion

Very little data exists in the literature regarding this condition. However, Chang Y-C et al., conducted a study of 2634 patients admitted for STEMI in which they identified the occurrence of hemoptysis in 11 patients, for an incidence of 0.4%^18^. Among our initially studied patients, we also found a proportion of approximately 4.5% presenting with alveolar hemorrhage after thrombolysis.

By including our two cases with the 23 others published in the medical English literature, we note that all patients were male and aged between 24 and 75 years [Table 1]. Both of our patients were diabetic and had a history of smoking [Table 2].

**Table 1:** Summary table of the 23 cases of alveolar hemorrhage following thrombolysis during myocardial infarction reported in the English medical literature.

**Author, Year**	**Age (Y)/** **Sex**	**Thrombolytic agent**	**Time interval**	**Underlying condition**	**Anemia**	**Hemoptysis**	**Infiltrate**	**Confirmation**	**Outcome**
Disler et al. 1990^4^	50/M	Streptokinase	5 days	Recent pneumonia	Yes	Yes	Bilateral		Recovered
Nathan et al. 1992^6^	52/M	Systemic r-TPA and intracoronary urokinase	24 h	Pulmonary catheterization, heart failure and PHT	Yes	Yes	Bilateral		Recovered
Obispo et al. 1992^7^	60/M	Streptokinase	36 h	CPR, defibrillation	Yes	Yes	Bilateral	COu	Recovered
Tio et al. 1992^8^	54/M	Streptokinase	3 days	Immune reaction	Yes	Yes	Bilateral		Recovered
Awadh et al. 1994^9^	63/M	Streptokinase	24 h	Heart failure	Yes	Yes	RUL, right upper lobe	Autopsy	Dead*
Hammoud et al. 1996^10^	70/M	r-TPA	12 h	Prior ipsilateral lung trauma 2 years earlier Defibrillation	Yes	Yes	RUL, right upper lobe		Recovered
Basher et al. 1996^11^	66/M	r-TPA	15 min	Right upper lung cavity of unknown etiology	No	Yes		Autopsy	Dead
Lee et al1997^13^	69/M	Urokinase	50 h	Pulmonary catheterization, pneumonia	Yes	Yes	Left lung		Recovered
Swanson et al. 1997^13^	58/M	Streptokinase	48 h	Immune reaction, COPD	Yes	Yes	Bilateral		Recovered
Gopalakrishnan et al. 1997^14^	24/M	r-TPA	24 h	Cardiac catheterization	Yes	Yes	Bilateral		Recovered
	64/M	r-TPA	12 h	COPD	Yes	Yes			Recovered
Masip et al. 1998^15^	65/M	Streptokinase	48 h	Immune reaction	Yes	Yes	Bilateral	LBA	Recovered
Yigla et al. 2000^16^	66/M	Streptokinase	48 h	Heart failure and PHT	Yes	Yes	Bilateral		Recovered
Ayyub et al. 2003^17^	35/M	Streptokinase			Yes	Yes	Bilateral	LBA	Recovered
Gonzalez et al. 0.2011^18^	42/M	Streptokinase	20 h			Yes	Bilateral		Recovered
Abuosa et al. 2014^19^	75/M	Streptokinase	72 h		Yes	Yes	Bilateral	LBA	Dead
Mahjoob et al. 2014^20^	45/M	Streptokinase		Cocaïne and Tobacco abuse	Yes	Yes	Bilateral	LBA	Dead
Narayanan et al. 2017^21^	58/M	Streptokinase	6 h		Yes	Yes	Bilateral		Recovered
Prasad et al. 2020^22^	65/M	Streptokinase	48 h		No	Yes	Bilateral		Recovered
	60/M	Streptokinase	6 h		yes	yes	Bilateral		Dead
Ben Mrad et al. 2021^23^	63/M	Streptokinase	24 h	Electrical cardioversion	Yes	Yes	Bilateral		Recovered
	61/M	Tenecteplase	2 days	Heavy smoker	Yes	Yes	Bilateral		Recovered
Mardenli et al. 2023^5^	64/M	Streptokinase	12 h	Bilateral chest trauma 10 months earlier	Yes	Yes	Bilateral		Recovered

**Table 2:** Comparative table of the two patients in our study.

**Patients** **Parametres**	**Case 1**	**Case 2**
Age/ Sex	71 yo / (M)	65 yo / (M)
Cardiac risk factors/ past medical history	– Type 2 Diabetes – Smoking – Sedentary life style – Overweight	– Diabetes type 2 – Smoking
Thrombolysis	Tenecteplase H5	Tenecteplase H2
Onset of symptoms	48 h	<24 h
Risk and underlying condition	DCCV performed for VT	– DCCV – Cardiac massage – Orotracheal intubation – Lung infection
Clinical signs	– Hemoptysis – Dyspnea	Hemoptysis
Chest X-ray	Alveolo-interstitial syndrom	Alveolo-interstitial syndrom
Chest CT-Scan	Diffuse ground-glass opacities, in the centrolobular regions	Not performed
Drop of hemoglobin	↓3.2g/dl	↓3.2g/dl
Bronchoalveolar lavage	Not performed	Not performed
Management	– Oxygenotherapy – Heparin discontinuation – Exacyl – Continuation of antiplatelet	Mechanical ventilation
Prognosis factors	– Inféro-latero- basal MI , LVEF = 50% – Sepsis	– Antérieur MI – Severe left ventricle dysfocntion (LVEF = 35%) – Cardiogenic Shock
Evolution	Death on day 5	Death on day 3

As described in the literature, the most patients were thrombolysed with streptokinase, a molecule implicated in the occurrence of autoimmune capillaritis. However, our two patients had received tenecteplase-based thrombolysis, which is not known to be implicated in the immune reaction causes of alveolar hemmorrhage^9,11– 29^. Thus, the cause suggested in our two patients might be the non-immune causes, such as vascular trauma secondary to the DVCC in the first case, and orotracheal intubation plus cardiac massage for the second one [Table 2].

Furthermore, in our two cases, hemoptysis appeared within 48 h for the first patient and in less than 24 h for the second [Table 2], similar to what is observed in the literature where bleeding occurred within 5 days of thrombolysis [Table 1]. This makes hemoptysis an important sign.

Regarding dyspnea, only our first patient presented with respiratory distress, while the second was intubated and ventilated as part of a cardiopulmonary arrest.

In terms of laboratory results, both of our patients had an identical drop in hemoglobin to 3.2 g/dL, which was the case for the majority of patients described in the literature [Tables 1 and 2].

On radiological examination, most patients had alveolar-interstitial infiltration, and we observed the same in our two patients. Note that, only the first patient in our study underwent a chest CT scan which showed typical ground-glass opacities, the second patient did not have a CT scan because he was hemodynamically unstable with acute kidney injury.

Bronchoalveolar lavage was not performed on any of our patients due to the limited benefit of this examination in predominantly unstable patients and, above all, the risk of bleeding after administration of a thrombolytic agent. In the literature, only 4 out of 23 patients underwent this examination to confirm the diagnosis [Table 1].

It should be noted that our patients had previously undergone risky procedures, both had coronary stenting and it is worth mentioning that the second patient had also undergone external cardiac massage with orotracheal intubation, this could probably explain the short time to onset of alveolar hemorrhage in this patient.

Currently, there are no international guidelines on the management of alveolar hemorrhage following thrombolysis in STEMI. The management of our patients was basically focused on oxygen therapy. Moreover, neither of the two patients was transfused because despite the drop in hemoglobin, they both had hemoglobin levels above 10 g/dL. Anticoagulation was discontinued in both patients, while dual antiplatelet therapy was continued, as they both had a newly implanted coronary stent, considering the ischemic risk which was as high as the bleeding risk.

Despite management, both of our patients died. The identified poor prognostic factors included pulmonary infection in the first patient and the extent of the myocardial infarction with cardiogenic shock in the second patient [Table 2].

In the literature, mortality rates range from 20 to 100%. Moreover, according to the 23 previously published cases, there was a mortality rate of approximately 21.73% [Table 2].

## Conclusion

Alveolar hemorrhage following thrombolysis in STEMI is a rare but potentially serious complication. It results from the rupture of small vessels in the pulmonary alveoli, facilitated by thrombolytic agents. Apart from the risk factors identified in our two cases such as male sex, history of diabetes and smoking, the direct current cardioversion delivery for arrhythmia or cardiopulmonary resuscitation measures, there are other risk factors for hemorrhage in the literature. This is a condition that can lead to acute respiratory failure and fatal hemorrhagic shock. Prevention relies on the careful selection of patients who are candidates for thrombolysis, as well as close monitoring of these patients after administration of thrombolytic therapy. Finally, primary angioplasty could be certainly an essential way to reduce the occurrence of this condition.
